# Effects of different exercise intensities of race-walking on brain functional connectivity as assessed by functional near-infrared spectroscopy

**DOI:** 10.3389/fnhum.2022.1002793

**Published:** 2022-10-14

**Authors:** Qianqian Song, Xiaodong Cheng, Rongna Zheng, Jie Yang, Hao Wu

**Affiliations:** ^1^Capital University of Physical Education and Sports, Beijing, China; ^2^School of Physical Education, Yanshan University, Qinhuangdao, China; ^3^School of Physical Education, Ludong University, Yantai, China

**Keywords:** functional near-infrared spectroscopy, race-walking, exercise intensity, brain activation, functional connectivity

## Abstract

**Introduction:**

Race-walking is a sport that mimics normal walking and running. Previous studies on sports science mainly focused on the cardiovascular and musculoskeletal systems. However, there is still a lack of research on the central nervous system, especially the real-time changes in brain network characteristics during race-walking exercise. This study aimed to use a network perspective to investigate the effects of different exercise intensities on brain functional connectivity.

**Materials and methods:**

A total of 16 right-handed healthy young athletes were recruited as participants in this study. The cerebral cortex concentration of oxyhemoglobin was measured by functional near-infrared spectroscopy in the bilateral prefrontal cortex (PFC), the motor cortex (MC) and occipital cortex (OC) during resting and race-walking states. Three specific periods as time windows corresponding to different exercise intensities were divided from the race-walking time of participants, including initial, intermediate and sprint stages. The brain activation and functional connectivity (FC) were calculated to describe the 0.01-0.1 Hz frequency-specific cortical activities.

**Results:**

Compared to the resting state, FC changes mainly exist between MC and OC in the initial stage, while PFC was involved in FC changes in the intermediate stage, and FC changes in the sprint stage were widely present in PFC, MC and OC. In addition, from the initial-development to the sprint stage, the significant changes in FC were displayed in PFC and MC.

**Conclusion:**

This brain functional connectivity-based study confirmed that hemodynamic changes at different exercise intensities reflected different brain network-specific characteristics. During race-walking exercise, more extensive brain activation might increase information processing speed. Increased exercise intensity could facilitate the integration of neural signals such as proprioception, motor control and motor planning, which may be an important factor for athletes to maintain sustained motor coordination and activity control at high intensity. This study was beneficial to understanding the neural mechanisms of brain networks under different exercise intensities.

## Introduction

Race-walking is a sport that mimics normal walking and running. The rules for this event, set by the International Association of Athletics Federations, require that the supporting leg remains straight at first contact with the ground until the body passes over the sole. In addition, the toes of the back foot should not leave the ground until the heel of the front foot touches the ground to ensure that both feet are not off the ground at the same time. In addition, the rules require the athlete to present a straightened knee from initial contact to the “vertical upright position” and no visible loss of contact. These rules distinguish race-walking from walking and running, making race-walking possesses a unique locomotor strategy different from other sports (Zhang et al., 2019). Physiological studies had shown that race-walking has higher restricted joint biomechanics and muscle energy costs than running (Cronin et al., 2016; Gomez-Ezeiza et al., 2019). Therefore, investigating the motor mechanisms of race-walking might be crucial for practitioners of this discipline.

The race-walking process is controlled by both peripheral and central systems. Traditionally, sports science focused on the effects of the exercise on cardiovascular and musculoskeletal systems. For example, the speed and distance of exercise is determined by the number of motor units that are recruited in our exercising limbs (Katz, 2010), the quality of the muscle fibers that produce force (Rae et al., 2010), and the size of the maximal cardiac output (Levine, 2008; Shephard, 2009). However, the theory that motor performance is primarily limited by the changes in cardiovascular and skeletal muscle metabolism has been generally questioned. With the fatigue development in the peripheral muscle fibers, the brain must regulate additional fresh fibers as compensation to assist those fatiguing fibers to sustain the work rate. This process continues progressively until all available motor units in the active muscle has been recruited. In fact, it has been established that less than 50% of active muscle fibers were recruited during prolonged exercise (Tucker et al., 2004; Amann et al., 2006); Even at maximum exercise intensity, that recruitment is only increased to about 60% (Albertus, 2008). Thus, the regulation of the central nervous system seemed to be the essence that determines the changes in motor performance.

There is growing evidence that the most important factors affecting motor performance begin and end in the brain (Noakes, 2011). Existing studies have described the relationship between the central system and the peripheral system through imaging methods. During incremental exercise, the concentration of oxygenated hemoglobin (HbO_2_) in the prefrontal cortex (PFC) continues to increase, and decreases before the end of exercise due to exhaustion (Rupp and Perrey, 2008; Kojima et al., 2022). Since reduced HbO_2_ in the PFC is associated with reduced muscle force generation capacity (Rasmussen et al., 2007), this may provide evidence that PFC plays a role in motor termination (Robertson and Marino, 2015). In addition, because of motor related areas could increase muscle power output with increasing exercise intensity, the neural activity of the motor cortex (MC) continues to increase in incremental exercise and it is also reported to decrease before the end of the exercise. However, some studies believe that no changes in neural activity seem to occur in PFC regions during submaximal and maximal exercise (Brümmer et al., 2011; Schneider et al., 2013), while changes in MC seem to be more able to reflect neural changes during movement (Jung et al., 2015). Over all, from the perspective of functional systems theory, the central nervous system determine the effectiveness of exercise (Kolomiets et al., 2017).

Recent research in neuroscience highlights the brain as a widespread and interconnected network that plays a fundamental role in controlling behavioral performance. Thus, analysis of regional activity levels may not adequately reflect the modulation of exercise-induced cortical mechanisms. The functional network throughout the cerebral cortex may be dramatically affected from normal resting to exercise states (Raichlen et al., 2016), and cortical network changes during exercise can be effectively evaluated in connectivity–based approach. Functional connectivity (FC) is defined as the statistical dependencies among remote neurophysiological events, which is a statistically quantifiable and observable phenomenon. Both activation and functional connectivity are parameters for evaluating the brain’s function (Stephan et al., 2009). A study of comparison on the effects of aerobic and anaerobic exercise on FC of brain network shows that aerobic exercise could increase the resting state FC in attention network (Schmitt et al., 2019). Furthermore, low and moderate-intensity exercise could enhance the resting state FC in the attention network as well as sensorimotor network (Rajab et al., 2014; Schmitt et al., 2019; Büchel et al., 2021). Thus, increased functional connectivity may contribute to the maintenance of exercise. In addition, the central executive network can be down-regulated if prolonged exercise is expected, in order to save mental resources (Radel et al., 2017). This might be another mode to the maintenance of exercise. However, how the functional interaction or connectivity changes between brain regions during a sustained high-intensity exercise remains a question that needed to be explored.

With the development of neuroimaging technology, it is possible to use functional near-infrared spectroscopy (fNIRS) to evaluate brain function changes during exercise in real time. The multichannel fNIRS instrument can measure the temporal correlation of cortical region changes and subsequently enable fNIRS-based activation or functional connectivity assessments (Chang-Hwan, 2010; Medvedev, 2014). The fNIRS is an optical imaging method based on the hemodynamic response, which can detect the changes in HbO_2_ and deoxygenated hemoglobin (HHb) concentration levels in the microcirculation of brain tissue very effectively, with good spatial and temporal resolution. (Jobsis, 1977; Naseer and Hong, 2015). Simultaneous functional magnetic resonance imaging (fMRI) research revealed that fNIRS measurement of HbO_2_ is highly correlated with fMRI measurement of blood oxygen level-dependent, suggesting that fNIRS could be an appropriate substitute for fMRI (Kim et al., 2017; Yücel et al., 2017). Compared with other non-invasive conventional functional imaging tools, such as fMRI and positron emission tomography, in general, fNIRS are characterized as safe, convenient, and inexpensive, and have fewer physical or environmental limitations and taboos (Naseer et al., 2014; Herold et al., 2017). Thus, it is readily applicable in the process of exercise to detect hemodynamic fluctuation in different movement states of athletes. In addition, filtering out cardiac and respiratory noise (Tachtsidis and Scholkmann, 2016; Fantini et al., 2018) can be addressed with the relatively high sampling rate of the fNIRS device (Fantini et al., 2018).

Performing high-intensity motor tasks might require a higher level of attention and sensorimotor processing to integrate visual and proprioceptive. Neuroimaging studies have shown that the PFC contributes to many higher-order behaviors, including motion planning, organization, regulation, speed, and direction of motion, and synthesis of multiple information required for complex behaviors (Hussar and Pasternak, 2013; Nee and D’Esposito, 2016). In addition, the PFC plays a broad role in controlling the process of goal-directed actions (D’Esposito et al., 2000; Miller and Cohen, 2001; Herd et al., 2006). The MC is mainly involved in the coordination and execution of complex motor sensations and motor functions, and controls human movement through spatial sensation and motor planning (Franceschini et al., 2003). The occipital cortex (OC), as visual cortex, is not only related to visual function, but also essential for conscious perception of various parts of the body, and can be modulated by visual stimuli, visually guided attention, and motor actions (Miller et al., 1993; Astafiev et al., 2004). These regions may be involved in responses to race-walking exercise in different coordinated ways. Thus, we measure the changes in cerebral oxyhemoglobin concentrations in the PFC, MC and OC. This study aimed to use a network perspective to investigate the effects of different exercise intensities on brain functional connectivity by the fNIRS imaging. We hypothesized that MC related brain functional connectivity would gradually increase to maintain the ability of the central system to regulate motor behavior in high exercise intensities. The results of this study might broaden our knowledge of the changes of brain functional networks during race-walking exercise. Real-time assessment of the functional status of central nervous system based on network connectivity characteristics helps to determine the strategy of the definite athlete adaptation, and estimates the effectiveness of the exercise process.

## Materials and methods

### Participants

Sixteen healthy young right-handed athletes aged 18–27 years from Ludong University were recruited to participate in this study. All of them met the competition criteria for the 2016 Rio de Janeiro Olympic Games (male 84-min and female 96-min for 20 km). Among the selected participants, 2 were excluded because of loose detectors during race-walking exercise. Therefore, the number of valid samples for the final analysis was 14 (8 males, accounting for 57%). All participants were screened for any history of significant medical, neurological or psychiatric injuries and disorders, which might affect brain structure or function. The researchers introduced the basic information (including experimental purposes, procedures, schedules, announcements, and contributions) of the experiment to the study participants and obtained their consent. All participants were required to have adequate sleep and were not allowed to participate in exercise within 24 h before the experiment. The Animal Ethics Committee of the Capital University of Physical Education and Sports (Beijing, China) approved all procedures and protocols. All participants signed informed consent before participating in all.

### Procedures

The experiment was conducted as a training lesson. Participants were asked to sit quietly for 5–10-min before the start of the experiment to eliminate the existing hemodynamic response caused by their activity. Subsequently, the research assistant wears the fNIRS equipment for the participants. The experiment was divided into two states, namely the resting state and race-walking state, and fNIRS was implemented continuously throughout the experiment. During the resting state, participants were instructed to stay awake with their eyes closed and remain quiet for 10-min. Then, all participants performed a 20-min warm-up. In race-walking state, participants were required to complete a 20-km race-walking on a treadmill (0% incline) within the required time of routine training. Three specific periods as time-windows corresponding to different exercise intensities were divided from the total time of participants. In order to better distinguish different exercise intensities, the three time-windows should be as far apart as possible. The fNIRS signal of 0.01–0.1 Hz was selected in this study, which was considered to be physiologically important and might reflect spontaneous neural activity (Scholkmann et al., 2014). In addition, more than five low-frequency periods are required to ensure the accuracy of the phase information (Bandrivskyy et al., 2004). Thus, the detection time for each time-window was set to 10-min. The first 10-min of race-walking state was extracted as the initial stage (male, 3.7 m/s; female 3.2 m/s), the middle 10-min as the intermediate stage (male, 3.8 m/s; female 3.3 m/s), and the last 10-min as the sprint stage (male, 4.0 m/s; female, 3.4 m/s). The detailed time schedule of race-walking was shown in Table 1.

**TABLE 1:** The detailed time schedule of race-walking.

Distance	Male			Female		
		
	Segmented time	Speed	Total time	Segmented time	Speed	Total time
0–2 km	9-min	3.7m/s	* 9-min	10-min 10-s	3.2m/s	*10-min 10-s
2–4 km	9-min	3.7m/s	* 18-min	10-min 10-s	3.2m/s	20-min 20-s
4–6 km	8-min 45-s	3.8m/s	26-min 45-s	10-min	3.3m/s	30-min 20-s
6–8 km	8-min 45-s	3.8m/s	35-min 30-s	10-min	3.3m/s	40-min 20-s
8–10 km	8-min 45-s	3.8m/s	^&^ 44-min 15-s	10-min	3.3m/s	^&^50-min 20-s
10–12 km	8-min 30-s	3.9m/s	^&^ 52-min 45-s	9-min 55-s	3.3m/s	^&^60-min 15-s
12–14 km	8-min 30-s	3.9m/s	61-min 15-s	9-min 55-s	3.3m/s	70-min 10-s
14–16 km	8-min 30-s	3.9m/s	69-min 45-s	9-min 55-s	3.3m/s	80-min 5-s
16–18 km	8-min 15-s	4.0m/s	^#^ 78-min	9-min 50-s	3.4m/s	^#^89-min 55-s
18–20 km	8-min 15-s	4.0m/s	^#^ 86-min 15-s	9-min 50-s	3.4m/s	^#^99-min 45-s

*Represent the start and end points of the initial stage’s time-window (male 0-min to 10-min, female 0-min to 10-min), ^&^represent the start and end points of the intermediate stage’s time-window (male 38-min 7.5-s to 48-min 7.5-s, female 44-min 52.5-s to 54-min 52.5-s), and ^#^represent the start and end points of the sprint stage’s time-window (male 76-min 15-s to 86-min 15-s, female 89-min 45-s to 99-min 45-s).

Basic information including gender, age, height, weight, blood pressure and medical history were recorded by the staff on the day before the experiment. Since heart rate measurement was a useful tool for detecting exercise intensity (Reis et al., 2011), the sports watch was used to monitor real-time heart rate changes during race-walking in the participants. The basic information and behavioral parameters of participants were shown in Table 2. In the present study, there were significant differences in the mean heart rates of the participants under the three time-windows, as shown in Table 3. The heart rate in each stage was averaged over the corresponding time-window. Therefore, the exercise intensity of the initial, intermediate and sprint stages could be distinguishable, respectively. During the race-walking, the experiment was terminated immediately if any symptoms developed or were suspicious (e.g., pain and tenderness, swelling, fever, redness or discoloration, and distension of the lower extremity surface veins, hypoxia, respiratory events, or chest pain).

**TABLE 2:** Basic information of participants and behavioral parameters.

Number	Age (year)	Gender	Education (year)	Height (cm)	Weight (kg)	Heart rate (b.p.m)			Blood diastolic/systolic pressure (mmHg)		Body temperature (°C)	
								
						Initial	Intermediate	Sprint	Pre	Post	Pre	Post
N1	20	Female	13	172	54	145	171	190	125/75	147/96	36.2	36.7
N2	21	Male	15	173	67	143	168	188	141/79	150/88	36.4	36.8
N3	25	Female	19	168	50	140	176	191	97/66	143/101	36.2	36.8
N4	27	Male	20	178	70	144	169	189	122/92	140/112	36.2	37.5
N5	18	Male	13	180	60	145	170	190	120/90	141/113	36.3	37
N6	21	Male	13	192	75	143	173	191	113/90	137/109	36.4	37.7
N7	25	Male	19	175	53	140	171	189	117/84	148/101	36.5	37.0
N8	25	Female	19	156	45	143	172	186	108/90	126/98	36.2	36.9
N9	24	Male	16	180	73	139	170	185	108/70	138/95	36.5	37
N10	21	Female	15	168	54	146	174	185	112/79	132/92	36.2	36.5
N11	19	Male	13	180	76	141	174	190	114/80	140/92	36.5	37
N12	19	Female	13	161	48	142	170	189	119/86	136/105	36.6	36.8
N13	20	Male	13	178	68	141	169	187	120/90	148/110	36.3	37.2
N14	20	Female	14	160	73	142	171	188	119/86	150/106	36.5	37.0

**TABLE 3:** Statistical analysis of participants in three time-windows.

Time-windows	Heart rate			
	
	Mean	Standard deviation	*P*-value	
Initial stage	142.43	2.10	<0.01	
Intermediate stage	171.29	2.27		<0.01
Sprint stage	188.43	2.03		

### Functional near-infrared spectroscopy data acquisition

**FIGURE 1:**
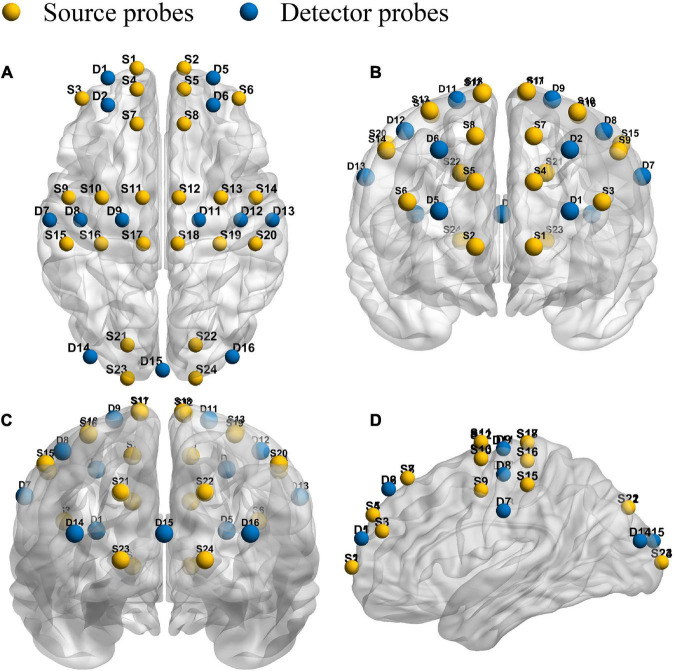
Schematic diagram of the fNIRS. Configuration of 24 source probes, 13 detector probes and 40 measurement channels. The top view **(A)**, the front view **(B)**, the rear view **(C)**, and the side view **(D)**.

This experiment adopts the multi-channel fNIRS system (NirSmart, Dan Yang hui gen medical instrument co., LTD.). In addition, the study adopted the 760 nm and 850 nm of two different wavelengths of near-infrared light to detect HbO_2_ and HHb concentration level changes. The inter–optode distance was 3 cm and the sampling rate was 10 Hz. Forty measurement channels, consisting of 24 light source probes and 13 detector probes, were established for measurement. The channel filling corresponding to 10/10 electrode positions was determined according to the different head sizes. With the help of the calibration function of the instrument and the corresponding template, the center of the middle probe setting row was placed near the FPz. As shown in Figure 1, the template and probe were symmetrically placed in the PFC (lPFC/rPFC), MCs (lMC/rMC), and OCs (lOC/rOC) regions on the left and right sides.

### Data pre-processing

The fNIRS signal preprocessing was carried out with the help of the NirSpark software package. During fNIRS, participants would have unavoidable head movements, especially during exercise. The commonly used correction methods for motion artifacts include spline interpolation, wavelet analysis, principal component analysis (PCA), etc., which had attracted extensive attention because of their great role in functional connectivity analysis (Zhang S. et al., 2021). In this study, the following steps were used to preprocess the fNIRS signals. In short, determining the components that may be associated with noise and artifacts requires us to perform PCA and independent component analysis (ICA) of the HbO_2_ and HHb signals for each channel. Firstly, PCA was used to reduce the dimension of each channel time series of each fNIRS, and ICA was used to separate the independent components of the same dimension. Through the inverse process of ICA and PCA, the remaining components of interest were reconstructed into signals of the same dimension as the original time series to separate unwanted sources from the hemodynamic response, including blood pressure, skin blood interference and non-evoked hemodynamic components (van de Ven et al., 2004; Zhang et al., 2010). Subsequently, the artifact portion was determined by identifying the impulse or cliff-type jumps caused by the relative sliding of the scalp and probes and was removed by cubic spline interpolation. Thirdly, the six–order Butterworth band–pass filter was used to retain the 0.01–0.1 Hz portion of the filtering signal and improve signal–to–noise ratio. Finally, the modified Beer-Lambert law was used to transform light intensity data into the relative change of the HbO_2_ and HHb based on different absorption spectra, the formula is as follows:


(1) $$O\,D^{\lambda \,i}=I\,n\,\frac{I_{O\,i}}{I_{i}}=\left(\varepsilon_{H\,B\,O}^{\lambda_{i}}\,C_{H\,B\,O}+\varepsilon_{H\,H\,B}^{\lambda_{i}}\,C_{H\,H\,B}\right)\,L_{\lambda_{i}}\,i=1,2$$

OD is the optical density, *I*_*Oi*_ is the intensity of incident light with a wavelength of λ*i*, *I_i_* is the corresponding scattered light intensity, and *L*_λ*_i_*_ is the optical path. $\varepsilon_{H\,B\,O}^{\lambda_{i}}\,C_{H\,B\,O}$ and $\varepsilon_{H\,H\,B}^{\lambda_{i}}\,C_{H\,H\,B}$ represent the light absorption coefficient and concentration of HbO_2_ and HHb with a wavelength of λ*i*, respectively. Δ*C*_*HbOHHb*_ represents the relative concentration changes in HbO_2_ and HHb concentrations. The following equation was used to calculate (Hu et al., 2019; Li et al., 2021):


(2) $$\Delta \,C_{H\,b\,O\,H\,H\,b}={\left(\begin{array}{c} \varepsilon_{H\,b\,O+H\,H\,b}^{\lambda_{1}}\\ \varepsilon_{H\,b\,O+H\,H\,b}^{\lambda_{2}} \end{array}\right)}^{-1}\,\left(\begin{array}{c} O\,D^{\lambda_{1}}/\left(r\times D\,P\,F^{\lambda_{1}}\right)\\ O\,D^{\lambda_{2}}/\left(r\times D\,P\,F^{\lambda_{2}}\right) \end{array}\right)$$

Studies had shown that HbO_2_ was the most sensitive marker of activity-dependent changes in regional cerebral blood flow (Dashtestani et al., 2018; Fang et al., 2018; Sulpizio et al., 2018), specially locomotion-related changes in cerebral oxygenation (Holtzer et al., 2015, 2016, 2017; Hernandez et al., 2016; Lin and Lin, 2016; Verghese et al., 2017). In addition, HbO_2_ had a superior contrast-to-noise ratio to HHb. Therefore, HbO_2_ signals were analyzed to describe the hemodynamic changes in this study.

### Brain activation

The cerebral oxygen signal measured by near infrared spectroscopy has very obvious time-frequency characteristics, and the frequency content can be continuously derived in time. By adjusting the length of the time domain and averaging the content in the time domain, the brain function parameters in a specific time range can be obtained. After preprocessing the original experimental data, a generalized linear model (GLM) was used to analyze the HbO_2_ signal time series data. GLM establishes the ideal hemodynamic response function (HRF) for each experimental paradigm and then calculates the degree of match between the experimental HRF value and the ideal HRF value, denoted by β. It represents the peak of the HRF function, and reflects the intensity or activation of the cerebral cortex (Chung et al., 2018; Ge et al., 2021). In this study, a pair of the adjacent light sources and detector forms a channel, and we calculate the intra-group average of *β-*value at the channel level. Then the image is generated by the interpolation method of inverse distance.

### Brain functional network connectivity

Through Pearson correlation analysis, the correlation coefficient between regions could be obtained and the FC intensity also could be studied (Ge et al., 2021; Zhang N. et al., 2021). Therefore, a 6x6 functional connectivity matrix could be calculated for each participant. The formula is as follows:


(3) $$r_{a,b}=\frac{c\,o\,v\,\left(a,b\right)}{\sigma_{a}\,\sigma_{b}}=\frac{E\,\left(a\,b\right)-E\,\left(a\right)\,E\,\left(b\right)}{\sqrt{E\,\left(a^{2}\right)-E^{2}\,\left(a\right)}\,\sqrt{E\,\left(b^{2}\right)-E^{2}\,\left(b\right)}}$$

*Cov*(*a,b*) represents the covariance of a and b; *E*(*a*) and *E*(*b*) represent the mean values of a and b, respectively; σ_*a*_ and σ_*b*_ represent the variance of *a* and *b*, respectively. *r*_*a,b*_ was used to evaluate the strength of FC. This indicates that there is a strong correlation between the two cortical regions, which is proportional to the *r*-value.

### Statistical analysis

In order to meet the assumptions required for parametric analysis, the normality test (Kolmogorov-Smirnov test) and variance uniformity test (Levene test) were performed on participant data, when *p* > 0.05, the variance was considered homogeneous. In this study, one–way ANOVA was performed on the region–wise activation and FC. Bonferroni correction was used for the multiple comparisons. There were six inter-group pair-wise comparisons, including resting vs. initial, resting vs. intermediate, resting vs. sprint, initial vs. intermediate, initial vs. sprint, and intermediate vs. sprint. The corrected *p*–value threshold was set at *p* < 0.0083 (0.05/6).

## Results

### Brain activation change

**FIGURE 2:**
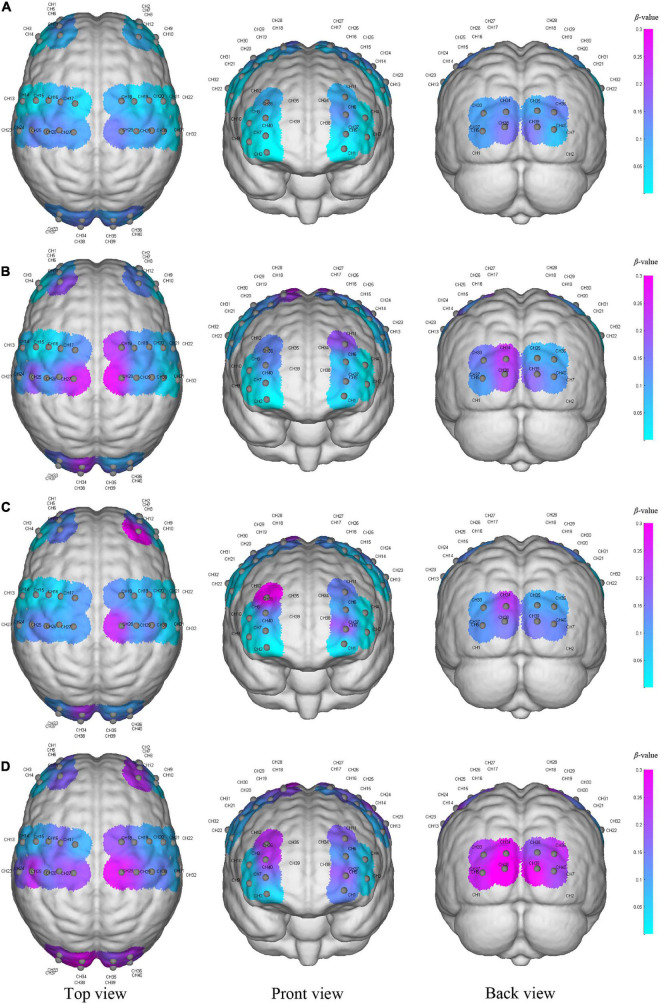
The activation of the cerebral cortex during resting state **(A)**, initial stage **(B)**, intermediate stage **(C)** and sprint stage **(D)** under three viewing angles (top view, front view, and back view). The color reflects the mean β-value of each region on the time scale, and the purple color represents higher activation than the blue–colored regions. Gray node represents the channel formed by each light source probe and detector probe, and the CH number is the label of the channel.

In this study, Figure 2 shows the results of cortical intensity or activation patterns in resting state (a), initial stage (b), intermediate stage (c) and sprint stage (d). The color bar number range on the right represents the *β-*value in the six brain regions and the purple color represents the higher activation than the blue color. We found that brain activation increased progressively with increasing walking intensity. In addition, the activation regions in the bilateral cerebral hemispheres were different during the initial, intermediate and sprint stages. However, although race-walking could induce a wide range of activation increases, there was no evidence that the *β-*value (*P* > 0.05) showed significant differences in the race-walking state compared with the resting state, as well as between different stages of race-walking.

### Functional connectivity change

**FIGURE 3:**
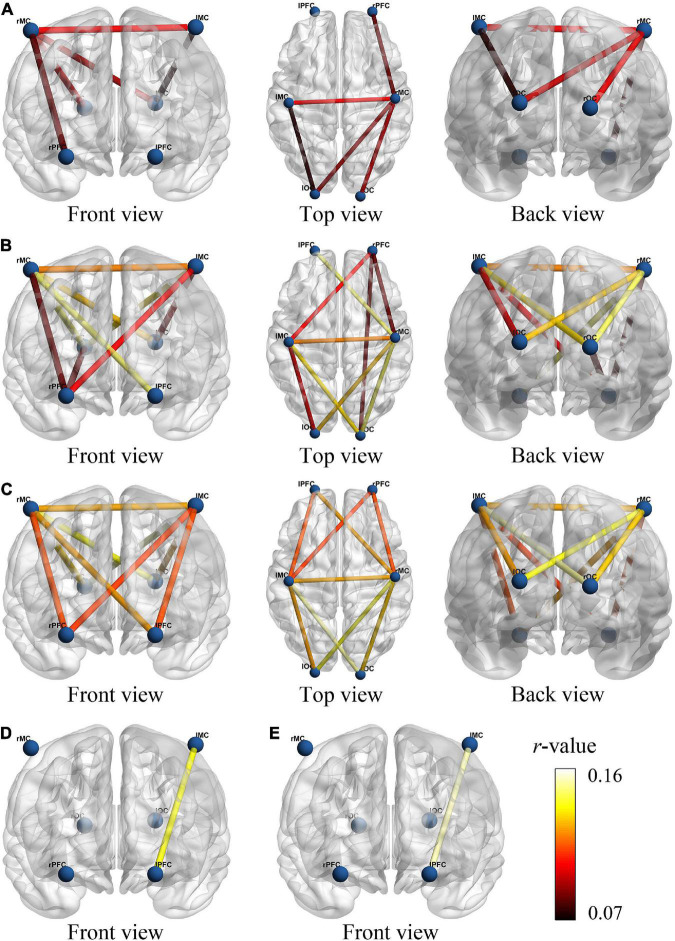
The functional connectivity visual map under different viewing angles. The connectivity line indicates the significant changes of *r*-value between resting and initial stage **(A)**, resting and intermediate stage **(B)**, resting and sprint stage **(C)**, initial and intermediate stage **(D)**, initial and sprint stage **(E)**. The blue nodes represent six brain regions. Line color indicates the connectivity intensity, and the brighter color represents higher strength.

We examined changes of FC values in different four states. Figure 3 provided a visual indication of the connectivity significantly change among the cerebral regions between any two states. The line color indicates connectivity intensity, blue dots indicate nodes. We found that the FC changes between resting vs. initial stage mainly exist between MC and OC, as shown in Figure 3A, while PFC was involved in FC changes between resting vs. intermediate as shown in Figure 3B, and resting vs. sprint were widely present in PFC, MC and OC as shown in Figure 3C. In addition, when the initial development to the sprint stage, the significant changes of FC were displayed in PFC and MC, as shown in Figures 3D,E.

**FIGURE 4:**
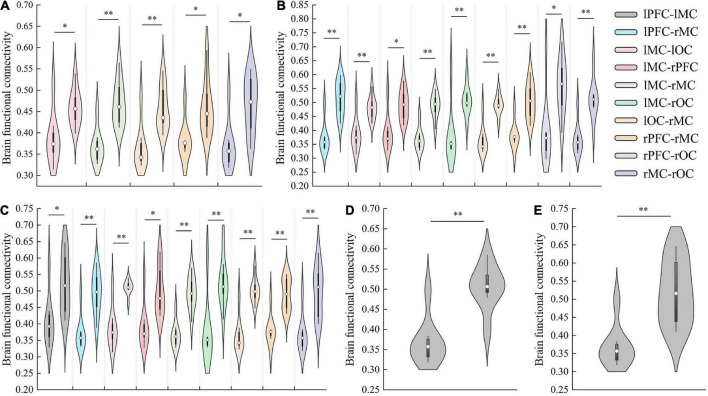
The results of significant changes of FC value between resting and initial stage **(A)**, resting and intermediate stage **(B)**, resting and sprint stage **(C)**, initial and intermediate stage **(D)**, initial and sprint stage **(E)**. *p < 0.05, **p < 0.01.

In detail, results of FC analysis showed that significant *r*-value increased in connectivity of lMC-lOC (*p* = 0.014), lMC-rMC (*p* = 0.001), lOC-rMC (*p* = 0.002), rPFC-rMC (*p* = 0.05), and rMC-rOC (*p* = 0.047), which was higher in initial stage than that in resting state, as shown in Figure 4A. Compared with resting state, significant increased FC value of lPFC-rMC (*p* < 0.001), lMC-lOC (*p* = 0.001), lMC-rPFC (*p* = 0.023), lMC-rMC (*p* < 0.001), lMC-rOC (*p* = 0.008), lOC-rMC (*p* < 0.001), rPFC-rMC (*p* = 0.001), rPFC-rOC (*p* = 0.016), and rMC-rOC (*p* = 0.001) were observed in intermediate stage, as shown in Figure 4B. The *r*-value exhibited significantly increased in lPFC-lMC (*p* = 0.022), lPFC-rMC (*p* = 0.001), lMC-lOC (*p* < 0.001), lMC-rPFC (*p* = 0.01), lMC-rMC (*p* < 0.001), lMC-rOC (*p* = 0.002), lOC-rMC (*p* < 0.001), rPFC-rMC (*p* = 0.003), and rMC-rOC (*p* = 0.004) in sprint stage compared with resting state, as shown in Figure 4C. In addition, the FC value of lPFC-rMC in intermediate stage (*p* = 0.004) and sprint stage (*p* = 0.001) were significantly increased than initial stage, as shown as Figures 4D,E.

Overall, FC results showed a continuous increase from the resting to the intermediate stage. However, an interesting result was that the FC between several regions, such as lPFC and rMC, decreased in the sprint stage. Moreover, there was no significant difference in FC was found between the sprint and intermediate stages.

## Discussion

In this study, portable fNIRS was used to detect the central nervous system activity during different time windows of race-walking, and cortical activation and FC indexes were used to explore the change patterns of brain network mechanisms under different intensity levels. The fNIRS signals were detected mainly in the 0.01–0.1Hz frequency interval, reflecting important neurovascular coupling activity (Scholkmann et al., 2014). This study mainly found that with the continuous development of race-walking, the mode of brain activation had undergone specific changes. In addition, the results of the brain network showed that with the exercise intensity rising, the intensity and number of brain connectivity began to increase significantly.

In this study, a seminal observation was that bilateral MCs activation occurred within 10 min of the beginning of race-walking compared to pre-exercise. Subsequently, hemodynamic responses of the prefrontal region were successfully induced, which might be the neural mechanism by which acute exercise could induce enhanced cognitive and control functions (Basso and Suzuki, 2017; Zaman et al., 2021). In the last 10 min of exercise, almost regional resources, including bilateral PFCs, MCs and OCs, participated in the sprint task. These discrepancies might reflect the following potential acute exercise effect. Our results suggested that spontaneous neural activity in PFC, MC and OC responds differently to exercise intensity. Spontaneous neural activity had been shown to have different physiological sources such as metabolic activity, neurogenic activity and myogenic activity in healthy people study (Xie et al., 2019, 2021), so hemodynamic parameters were closely controlled and regulated by these factors. Some neuroimaging reports believed that cerebrovascular reactivity was associated with chronic and acute exercise, respectively (Svensson et al., 2015; Vilela et al., 2017; Mueller et al., 2020; Vints et al., 2022), which also supports our conclusions. At higher exercise intensity, extensive cortical activation might improve information processing speed (Chang et al., 2012). In addition, the regional allocation of cerebral blood flow during the exercise stage might reflect changes in functional connectivity since others report acute exercise impacts attention-related brain networks (Kamijo et al., 2004; MacIntosh et al., 2014).

The quantification of cortical activation was a theoretical analysis based on the functional separation model of brain regions, while in reality cortical regions appeared as a brain network closely related in structure and function. The exercise was traditionally considered to be a process involving motor-related brain regions, such as the primary motor and premotor areas. However, the movement also involves many non-motor regions (Rizzolatti et al., 2014). In previous studies, the focus on the activation of brain regions and cortical activation changes during exercise was one-sided. The brain functional network method based on quantifying the number and strength of brain connectivity might have more authenticity.

Our FC results showed that MC-related brain networks first undergo specific changes at the early stage of exercise, such as a significant increase in the information exchange between bilateral MCs. Recent neuroimaging studies have shown that task-evoked functional connectivity was thought to have transient interactions between specific brain regions related to specific task performance (Moore et al., 2022), and exercise could enhance connectivity in sensorimotor-related brain networks (Rajab et al., 2014). We believe that this increase in the level of homologous interbrain interaction was related to the efficiency of effective information transmission, which might be beneficial to further strengthen the control and planning of movement, and this notion has been confirmed by human and rodent studies (Venezia et al., 2017; Moore and Loprinzi, 2021).

As exercise intensity increases, the connectivity between bilateral MCs and other regions began to strengthen, such as bilateral OCs. These results suggested that race-walking could induce enhanced synchrony of neural activation in bilateral MC and OC. On the one hand, this result was consistent with the conclusion that motor performance depended more on bilateral MCs when tasks were more demanding (Verstynen and Ivry, 2011). On the other hand, performing more complex motor tasks required higher levels of visuomotor coordinated responses, and OC might play a role in the integration of body parts perception and sensorimotor functions (Astafiev et al., 2004).

In addition, the connectivity between PFC and MC changed most obviously in the sprint stage, and the main difference between the sprint stage and the starting stage also existed in these regions. This phenomenon was also confirmed by a recent study, which showed that increased exercise intensity could induce positive connectivity between the frontal network system and other cortices, especially between the PFC and the parietal lobe (Voss et al., 2020). The PFC and MC were important components of the fronto-parietal motor control network, which involve cognitive and control functions. Generally, PFC is a major hub of default mode network and executive control network (Buckner et al., 2008), and had basic functions such as imagination and emotion processing. Recent neuroimaging studies had shown that apart from being important in cognitive function, PFC accomplishes motion planning, organization, regulation, speed, and direction of motion (Hussar and Pasternak, 2013; Nee and D’Esposito, 2016). Emerging evidence from human behavioral experiments suggested that acute exercise was associated with improved performance in the PFC, which determines executive ability during motor tasks (Astafiev et al., 2004; Guiney and Machado, 2013). Therefore, we had reasonable speculation that when exercise intensity reached a certain level, the strength of functional connectivity between the prefrontal lobe and the motor region was the main neural mechanism that affected exercise ability and performance.

It should be noted that only right-handed participants were selected for this study. Studies have shown that handedness could affect individual brain function, including activation regions and network connectivity (Lee et al., 2019; Nair et al., 2019), so the conclusions of this study might not be fully applicable to left-handed athletes. In addition, our study has several limitations. Firstly, short-distance channels were not used in the present study, which might be one of the most effective methods to purify the signal. The fNIRS signal mainly contains evoked components and non-evoked components (Scholkmann et al., 2014). We currently use PCA, ICA and an effective preprocessing method could remove non-evoked components and part of the evoked components (cannot distinguish evoked cerebral functional brain activity from evoked cerebral systemic activity). Therefore, short-distance channels should be used as a standardized step in future research. Another limitation is that we did not use wearable tools to monitor participants’ peripheral skeletal muscle data during race-walking, such as electromyography. We believe that because the fNIRS was implemented continuously throughout the experiment, the wearing of other monitoring devices for participants will increase the physiological burden, which could cause the negative effects on the brain network. Therefore, the focus of this study was to investigate changes in brain networks, rather than central-peripheral or neuromuscular coupling during race-walking. Thirdly, due to the characteristics and limitations of fNIRS, we only monitored cortical hemodynamic changes during race-walking. However, some subcortical regions, such as the insula, thalamus, and basal ganglia, are also involved in the neural control of motor performance (Moore et al., 2022). Therefore, more interesting results might be obtained by combining deep neuroimaging tools based on the methods in this study.

Overall, this network connectivity-based study confirmed that hemodynamic changes at different exercise intensities reflected different brain network-specific characteristics. Spontaneous neural activity in the PFC, MC, and OC responded differently to exercise intensity, and more extensive brain activation might increase information processing speed. In brain network connectivity, the increase in exercise intensity could improve the functional interaction between brain regions, which will be beneficial to integratng neural signals including proprioception, motor control and motor planning. It might be an important factor for athletes to maintain continuous motor coordination and control of activities under high intensity. This study extended the understanding of the brain networks involved in different exercise intensities, and it could be used as a sensitive and effective neural parameter to evaluate the effects of exercise.

## Data availability statement

The raw data supporting the conclusions of this article will be made available by the authors, without undue reservation.

## Ethics statement

The studies involving human participants were reviewed and approved by the Institutional Animal Ethical Committee of the Capital University of Physical Education and Sports, Beijing, China. Written informed consent to participate in this study was provided by the participants’ legal guardian/next of kin. Written informed consent was obtained from the individual(s), and minor(s)’ legal guardian/next of kin, for the publication of any potentially identifiable images or data included in this article.

## Author contributions

QS, XC, RZ, JY, and HW worked together to complete the manuscript. QS, JY, and HW contributed to the conception and design of the study. QS and JY carried out the experiments. QS performed the data analyses and wrote the manuscript. QS and RZ provided statistical assistance and support. XC, JY, and HW provided opinions on grammar and rhetoric. All authors contributed to manuscript revision, read, and approved the submitted version.
